# Nanomedicine in Cardiovascular Inflammation: Novel Diagnostic and Therapeutic Strategies

**DOI:** 10.3390/jpm16060328

**Published:** 2026-06-18

**Authors:** Aikaterini-Eleftheria Karanikola, Agapi Ploussi, Dimitrios Tsiachris, Efstathios P. Efstathopoulos

**Affiliations:** 1 Medical School, National and Kapodistrian University of Athens, 11527 Athens, Greece; elinakaranikola@gmail.com (A.-E.K.); dtsiachris@yahoo.com (D.T.); 2 Department of Applied Medical Physics, Medical School, National and Kapodistrian University of Athens, 12462 Athens, Greece; aplousi@gmail.com

**Keywords:** cardiovascular inflammation, nanomedicine, theranostics, precision medicine, personalized medicine

## Abstract

Inflammation plays a central role in the pathogenesis and progression of cardiovascular diseases, including atherosclerosis, myocardial infarction and heart failure. Despite advances in conventional diagnostic and therapeutic strategies, limitations in sensitivity, specificity and targeted drug delivery still remain. Nanomedicine has emerged as a promising yet underexplored approach to address these challenges by enabling precise molecular imaging and site-specific therapeutic interventions. This review summarizes current and emerging nanotechnology-based approaches for the diagnosis and treatment of cardiovascular inflammation, highlighting their potential in clinical practice and remaining challenges. In addition, recent advances, including the development of biomimetic nanoplatforms, are discussed, along with future perspectives and the potential integration of artificial intelligence to further enhance precision in cardiovascular medicine.

## 1. Introduction

In recent years, inflammation has been recognized as a key pathophysiological mechanism of multiple cardiovascular diseases (CVD), including atherosclerotic CVD (ASCVD), thrombosis, coronary artery disease (CAD), heart failure (HF), pericarditis and atrial fibrillation (AF) [1]. Several biomarkers have been strongly associated with CVD development and progression, including fibrinogen, interleukin-6 (IL-6), C-reactive protein (CRP) and galectin-3 [2]. In addition, increasing evidence supports a pivotal role for the NOD-like receptor pyrin domain-containing 3 (NLRP3) inflammasome in CVD. NLRP3 is expressed in circulating leukocytes as well as in myocardial, endothelial, and vascular smooth muscle cells, and its activation via pro-inflammatory cytokines and NF-κB signalling has been implicated in multiple conditions, including ASCVD, hypertension, myocardial infarction (MI), thrombosis, dilated cardiomyopathy, and HF, among others [3].

Despite advances in understanding of the mechanistic background, the incorporation of anti-inflammatory strategies into routine cardiovascular care remains limited. The importance of clinician awareness and evidence-based implementation of anti-inflammatory therapies is emphasized in the recent American College of Cardiology scientific statement on inflammation and cardiovascular disease, which outlines key inflammatory pathways and therapeutic targets. However, this report largely focuses on traditional pharmacological approaches and does not address the emerging, potentially promising role of nanomedicine in the management of cardiovascular inflammation [4].

Nanomedicine refers to the application of materials at the nanoscale, typically 1–100 nm, for diagnostic, therapeutic, or combined theranostic purposes. Owing to their particularly small size and unique physical, chemical, and biological properties, nanoparticles (NPs) can be engineered to enable disease diagnosis through precision biosensing platforms, enhance biodistribution, and facilitate targeted drug delivery [5,6].

Nanotechnology-based diagnostic strategies have emerged as promising tools for detecting cardiovascular inflammation, surpassing the limitations of conventional imaging approaches. NPs can be engineered to accumulate at sites of inflammation and to interact with specific cellular and molecular targets, thereby enabling highly specific molecular imaging. Additionally, the unique physicochemical characteristics of nanomaterials make nanoplatforms particularly attractive for overcoming key limitations of traditional cardiovascular therapies, such as poor tissue specificity, suboptimal bioavailability, and systemic adverse effects, thus making them ideal for site-specific drug delivery and controlled release. In addition to targeted therapeutic drug delivery, another significant advantage of nanosystems is their theranostic potential, that integrates both diagnostic and therapeutic functions within a single nanoplatform, enabling simultaneous imaging, disease monitoring, and treatment [7].

This comprehensive review aims to highlight the central role of inflammation in major cardiovascular diseases and summarize current and emerging nanotechnology-based approaches for the diagnosis and treatment of cardiovascular inflammation, with a particular emphasis on targeted nanoplatforms for imaging and drug delivery.

## 2. Methods

The literature for this narrative review was identified through PubMed, Scopus and Web of Science using Boolean operators and combinations of terms such as “nanomedicine”, “cardiovascular inflammation”, “atherosclerosis”, “myocardial infarction”, “heart failure”, “cardiomyopathy”, “atrial fibrillation”, “myocarditis”, “pericarditis” and “theranostics”. Articles published in English between 2021 and 2026 were primarily considered, with older landmark studies included when considered relevant and highly contributory to the field. Both original research and review articles were screened to provide a comprehensive overview of current evidence. Titles and abstracts were independently screened by two authors, and citation tracking (snowballing) was used to identify additional relevant studies. All references were managed in a reference manager.

The review focuses on studies most relevant to the diagnostic and therapeutic nanoplatforms targeting inflammation across a wide spectrum of cardiovascular diseases and aims to summarize current research and provide future directions. Given the broad and evolving nature of the topic, this work was conducted as a narrative rather than a systematic review; therefore, a PRISMA-based selection strategy was not applied. Due to this limitation, selection bias and incomplete literature inclusion cannot be fully excluded.

## 3. Nanotechnology-Based Approaches in Cardiovascular Inflammation

**Figure 1:**
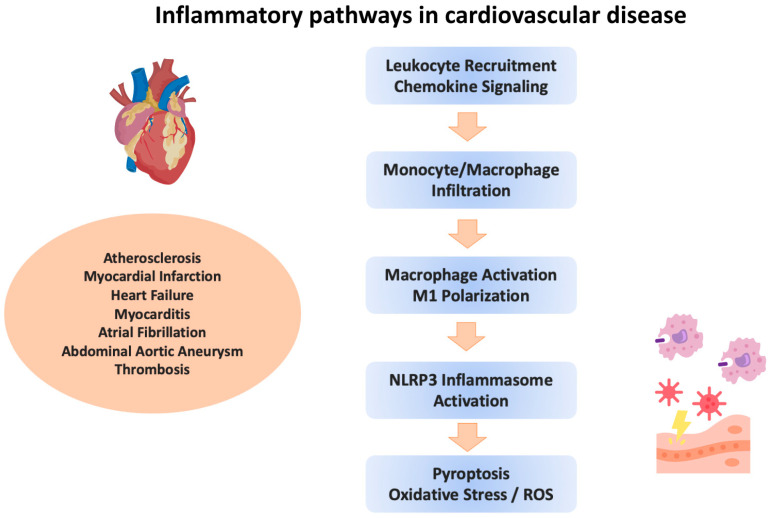
**Main inflammatory mechanisms involved in cardiovascular disease**. Inflammation plays a central role in the initiation and progression of several cardiovascular diseases, including atherosclerosis, myocardial infarction, heart failure, myocarditis, atrial fibrillation, abdominal aortic aneurysm, and thrombosis. Following an inflammatory trigger, leukocyte recruitment and chemokine signaling promote monocyte and macrophage infiltration into cardiovascular tissues. Subsequent macrophage activation, particularly toward a pro-inflammatory phenotype M1, contributes to activation of the NLRP3 inflammasome, leading to pyroptosis, oxidative stress, and the production of inflammatory mediators. These processes collectively drive tissue injury, adverse remodeling, and disease progression, highlighting key inflammatory pathways that may serve as potential diagnostic and therapeutic targets.

In the following sections, the main inflammatory mechanisms (Figure 1) across major cardiovascular disease entities, as well as current nanotechnology-based approaches targeting cardiovascular inflammation will be summarized. Table 1, Table 2 and Table 3 include examples of nanoplatforms used for diagnosis, therapy and theranostics, including the level of evidence of the respective nanosystems. Although numerous nanoplatforms have demonstrated promising results in experimental settings, the level of evidence varies considerably across cardiovascular conditions, with the strongest translational data currently available in atherosclerosis and myocardial infarction, whereas applications in heart failure, cardiac arrhythmias, and myopericardial diseases remain largely exploratory.

### 3.1. Atherosclerosis and Plaque Vulnerability

Beyond traditional risk factors, chronic low-grade inflammation contributes to endothelial dysfunction, lipid accumulation, and vascular remodeling, promoting atherogenesis and plaque instability in ASCVD. Pro-inflammatory cytokine-driven macrophage activation promotes metalloproteinase release, reduced LOX activity, cell apoptosis, and microcalcifications, leading to weakening of the plaque fibrous cap and eventually to rupture or erosion, causing acute myocardial infarction (MI) [60]. Considering the key role of macrophages in the progression of ASCVD, these cells have been identified as potential diagnostic and therapeutic targets.

Several nanoplatforms have been developed as contrast agents for various non-invasive imaging modalities—including computed tomography (CT), magnetic resonance imaging (MRI) and positron emission tomography (PET)—to detect atherosclerotic plaques. Clinically, superparamagnetic iron oxide nanoparticles (SPIONs) and ultra-small superparamagnetic iron oxide nanoparticles (USPIONs) have long been utilized in molecular MRI to assess plaque burden and monitor therapeutic response, as their uptake by plaque macrophages induces signal loss in T2-weighted images, thereby enhancing plaque contrast [8,9,61]. Both probes demonstrate a favorable safety profile compared with conventional gadolinium(Gd)-based contrast agents, even in patients with renal dysfunction, and USPIONs in particular may offer even better imaging potential due to their prolonged circulation time [9]. Additionally, Gd-based paramagnetic contrast agents conjugated with NPs can specifically target plaque macrophages. For instance, crosslinked hyaluronic acid NPs (cHANPs) exhibit increased hydrodenticity, producing greater T1 reduction than free Gd-DTPA and resulting in improved imaging precision and potentially reduced systemic toxicity [10]. Other researchers developed Gd-containing amorphous calcium carbonate NPs ligated with trimannose to achieve effective macrophage targeting and enhanced contrast for lesion characterization in MRI images, thereby supporting noninvasive assessment of inflammatory burden in ASCVD [11]. In CT imaging, NPs engineered with iodixanol and hydrophobic moieties have effectively targeted macrophages within atherosclerotic lesions, enabling high-sensitivity lesion detection with low toxicity in animal models [12]. Moreover, gold NP–based imaging probes, combined with annexin V and labelled with Technetium-99m, have been used in SPECT/CT to identify vulnerable atherosclerotic plaques by targeting apoptotic cells [13]. Other researchers, recognizing the central role of chemokines in atherosclerosis initiation and progression, developed a radiolabeled vMIP-II–conjugated NP targeting multiple macrophage-associated chemokine receptors, enabling sensitive and specific PET/CT imaging of plaque inflammation and progression in a mouse model [14]. Specifically, chemokine receptor CCR5, a key molecule in plaque development and vulnerability, has been targeted with 64Cu-DAPTA-Comb NPs, enabling precise in vivo PET/CT detection of CCR5 expression that correlates with lesion progression and regression [15].

Although NPs have several applications in non-invasive atherosclerosis imaging, their use in intravascular imaging has been limited. These approaches are focused mainly on established techniques, such as intravascular ultrasound (IVUS) and optical coherence tomography (OCT), often in combination with near-infrared spectroscopy (NIRS) or fluorescence (NIRF) [62]. For instance, Kee et al. produced anti-ICAM-1–conjugated echogenic liposomes and further enhanced these with nitric oxide. The resulting microbubbles exhibited enhanced echogenicity and plaque-targeting by increasing vascular permeability, enabling improved molecular imaging of early atherosclerosis with IVUS [16]. In a recent study by Chen et al., a dual-modality OCT-fluorescence imaging contrast agent enabled the detection of porphyrin-HDL NPs that accumulate in macrophage-rich plaques in atherosclerotic mice, thereby enabling early disease detection. These findings were validated by histological analysis and fluorescence microscopy, highlighting the probe’s ability to capture both structural and molecular plaque changes [17]. In another study, a contrast agent based on infrared-emitting semiconductor quantum dots (QDs) enabled simultaneous OCT and fluorescence imaging, providing multimodal intracoronary imaging and highlighting the potential of these specific NPs for advanced plaque characterization [18]. The use of nanomaterials in other intravascular imaging methods, such as Photoacoustic Imaging (PAI), has demonstrated promising results. However, no commercially available PAI devices have been developed to this day [63].

In contrast to purely therapeutic nanoplatforms, theranostic nanosystems combine diagnostic imaging capabilities with targeted therapeutic delivery within a single platform. Inflammation-targeted theranostic nanomedicine represents a promising approach for atherosclerosis management, enabling simultaneous real-time molecular imaging and site-specific drug delivery. For example, polydopamine NPs doped with arginine and Gd ions delivering miR-146a to inflammatory macrophages provide intrinsic T1 MRI contrast while simultaneously eliminating ROS and suppressing NF-κB-mediated inflammation, thereby reducing and stabilizing plaques [54]. Similarly, dual-responsive nanoplatforms utilize ROS/MMP-triggered degradation to release anti-inflammatory agents precisely at inflamed lesions while enabling PAI for plaque recognition [55]. Other researchers formulated a macrophage mannose receptor–targeted theranostic nanodrug that delivers the PPARγ agonist lobeglitazone and inhibits the TLR4/NF-κB pathway. This strategy enabled simultaneous detection and stabilization of inflamed high-risk plaques to a macrophage-depleted, collagen-rich type, as shown by reduced inflammation on OCT-NIRF imaging in atheromatous rabbits [56]. By integrating diagnosis with targeted immunomodulation, theranostic nanomedicine offers a precision approach to identify vulnerable plaques and deliver site-specific anti-inflammatory interventions at an early stage of disease, potentially preventing acute cardiovascular events.

### 3.2. Myocardial Infarction and Ischemic Injury

Inflammation in acute myocardial infarction (AMI) is a dynamic process with varying contributions across different stages of disease progression. An intense early inflammatory response, driven by neutrophil and monocyte activation and a rise in pro-inflammatory cytokines following plaque rupture and thrombosis, is thought to promote myocardial repair but, when in excess, to further contribute to ischemic myocardial injury. In contrast, chronic low-grade inflammation in later stages promotes adverse left ventricular remodeling and is associated with poorer clinical outcomes [64]. Key drivers of this inflammatory response include M1 (pro-inflammatory) macrophage polarization, NLRP3 inflammasome activation, and increased interleukin production [64]. Inflammasomes also activate the caspase pathway, eventually leading to pyroptosis, a form of programmed cell death that is increasingly recognized as a major player in the post-MI inflammatory state [65].

A major application of nanomedicine in the diagnosis of MI is targeted molecular imaging of inflammatory activity in the infarct zone. NP-based probes can be engineered to target the inflammatory cascade and directly visualize it with various imaging modalities. For example, NP-contrast agents have been used to detect apoptosis, fibrin deposition, and other inflammatory markers in ischemic myocardium, helping identify damaged tissue with increased accuracy and precision, potentially enabling timely intervention. Clinically, USPIONs, such as ferumoxytol, represent the most established platform for MRI-based visualization of inflammatory activity in MI, with early clinical studies demonstrating feasibility and safety [19,20]. In nuclear imaging, macrophage-targeting probes such as Macroflor enable quantitative PET imaging of cardiac macrophage burden, while cross-linked iron oxide NPs provide multimodal MRI–optical imaging of CCR2+ myeloid cell dynamics, with early imaging signatures correlating with long-term functional outcomes [21,22]. These NP-based approaches may offer advantages over conventional ^18^F-FDG PET, which often yields false-positive results due to myocyte glucose uptake in ischemic tissue, and hold promise for guiding anti-inflammatory therapies by identifying patients with excessive inflammatory responses. Regarding optical imaging, its widespread use in preclinical studies is not reflected in the clinical setting. However, several imaging nanoprobes have been developed for PAI, fluorescent and near-infrared (NIR-II) imaging [66]. For example, angiotensin II-functionalized Ag_2_S nanodots enable selective NIR-II imaging of ischemic myocardium by targeting upregulated AT1 receptor [23]. In parallel, multimodal nanotracer systems integrating ^19^F-MRI, PET, and optical imaging enable comprehensive tracking of myeloid cell activity in the circulation and myocardial tissue, providing valuable insights into systemic inflammatory responses following MI [24]. Additionally, some researchers have investigated nanoplatforms for combined imaging modalities, including the use of functionalized MnO-PEG-Cy5.5 NPs, which provide dual MRI and NIR imaging capabilities and high accumulation in the infarcted area, potentially making them promising vehicles for site-specific drug delivery [25].

Beyond diagnostics, nanomedicine has increasingly been applied to modulate post-MI inflammation and ischemia–reperfusion (I/R) injury, a key determinant of infarct size and remodeling. Although the association of post-MI inflammation with poorer clinical outcomes and increased mortality has been established, traditional and novel anti-inflammatory therapies targeting the inflammasome and interleukin activation are currently being tested, with inconsistent efficacy and safety outcomes [67]. Recently, nanomaterials have been used to modulate the inflammatory response post-MI and suppress ischemic injury, owing to their unique properties and therapeutic potential, as suggested by evidence from animal studies [68]. Several nanomedicine-based approaches target the mechanism of cardiomyocyte pyroptosis by delivering anti-inflammatory agents that inhibit key pathways, such as NLRP3–caspase-1 signaling, thereby reducing inflammation and promoting cardiac repair [32]. Colchicine, a widely used anti-inflammatory agent recommended in clinical guidelines as adjunctive therapy for cardiovascular risk reduction following MI, has also been effectively delivered via inorganic NP-based platforms. In vivo studies have demonstrated that such delivery systems can significantly reduce infarct size by nearly 45%, reduce residual fibrosis, and lower serum inflammatory biomarkers [33,69].

Inflammation also plays a central role in the pathophysiology of ischemia–reperfusion (I/R) injury and therefore exhibits both diagnostic and therapeutic potential. Various drug delivery systems have been formulated to attenuate I/R injury after thrombolysis or coronary revascularization by targeting oxidative stress, inflammation and apoptosis [70]. For instance, Guo et al. developed a platelet membrane-coated, thrombus-targeting nanomedicine that enables targeted delivery and controlled release of tPA to dissolve thrombi during ischemia, while simultaneously eliminating reactive oxygen species (ROS) during reperfusion and preserving mitochondrial function [34]. Among the nanomaterials employed in targeted antioxidant delivery platforms, metal–organic frameworks (MOFs) and liposomes have been investigated most extensively. These organic and inorganic NPs have shown effective and safe drug delivery, leading to enhanced antioxidant and anti-inflammatory effects, reduced cardiomyocyte apoptosis, and overall improvements in cardiac function and remodeling [35,36]. Finally, suppression of NLRP3 inflammasome activation plays a crucial role in preventing I/R injury, and preclinical studies have demonstrated the potential of transferrin-conjugated polymeric nanomicelles for targeted delivery of inflammasome inhibitors to occluded arteries [37].

More recently, nanotheranostic platforms integrating both imaging and targeted therapeutic delivery have emerged as promising investigational tools for MI management. Hybrid systems such as HM4oRL combine immune membrane coating, bioactive lipid-loaded liposomes, and metal–polyphenol networks to simultaneously suppress cardiomyocyte pyroptosis, reprogram macrophage polarization, and enable T1-weighted MRI-based monitoring of inflammatory status during the initial stages of MI [57]. Additionally, perfluorocarbon NP systems delivering thrombin inhibitors such as PPACK directly at an occluded coronary artery under visualization with fluorescent microscopy and 19F MRI, have exhibited both local thrombin suppression and a reduction in inflammation, microvascular injury and I/R injury, with simultaneous preservation of cardiac systolic function [58].

### 3.3. Heart Failure and Cardiomyopathies

#### 3.3.1. Chronic Heart Failure and HFpEF

Heart failure (HF) is the clinical syndrome resulting from impaired systolic and/or diastolic function of the myocardium. The etiology behind these structural and/or functional cardiac changes is diverse and includes, among others, coronary artery disease, hypertension, valvular heart disease, arrhythmias, cardiomyopathies, drug-induced and infectious causes [71]. Several inflammatory mechanisms have been implicated in the pathogenesis of HF. Increasing evidence suggests that HF represents a chronic systemic inflammatory state characterized by persistent immune activation that contributes to disease progression and adverse outcomes independently of traditional clinical parameters. Inflammatory biomarkers, including CRP and IL-6 have been consistently associated with increased disease severity, hospitalization, and mortality, further highlighting inflammation as a promising therapeutic target in HF [72]. Notably, interleukin-1 (IL-1) signalling contributes to both systolic and diastolic dysfunction by disrupting calcium metabolism, leading to congestion, hypoperfusion, and subsequent neurohormonal activation, thereby promoting HF progression and persistence [73]. In addition, the chronic proinflammatory state associated with CVD comorbidities may trigger endothelial inflammation, impaired nitric oxide (NO) production, and oxidative stress, which, in turn, promote microvascular dysfunction, diastolic dysfunction and HF with preserved ejection fraction (HFpEF) [74]. Other mediators of inflammation in HF include pro-inflammatory cytokines, such as tumor necrosis factor alpha (TNF-α) and IL-6, immune cells and particularly macrophages, angiotensin II (Ang II) and Myeloperoxidase (MPO) [75].

The use of nanostructures in HF therapeutics has been supported by the hypothesis that inflammation leads to vascular injury and endothelial dysfunction, resulting in enhanced permeability and retention (EPR) of failing myocardial cells [76]. In addition, several of the diseases ultimately leading to HF are highly inflammatory processes and targeting them in their early stages may prevent cardiac function deterioration, as evidence from preclinical studies has shown. For example, NO-releasing hydrogel NPs (NO-NPs), a combination of SPIONs and a NO-precursor, have exhibited their potential as an effective nanotherapy for endothelial dysfunction and cardiac injury in a zebrafish HF model. By enabling sustained NO release, these NPs improved survival, restored cardiac contractility, and reduced inflammation by downregulating IL-6 and COX-2 expression [40].

#### 3.3.2. Chemotherapy-Induced Cardiomyopathy

Cardiotoxicity from chemotherapeutic agents, particularly anthracyclines, such as doxorubicin, is an increasingly recognized cause of HF, with inflammation and oxidative stress being central to doxorubicin-induced cardiomyopathy, driving immune cell infiltration and cardiac injury [77]. A combination of anti-inflammatory and antioxidant therapy has shown great promise in preventing drug-induced HF. Liu et al. developed ROS-scavenging TPCD NPs paired with Ac2-26, an annexin A1 N-terminal-derived peptide with anti-inflammatory properties, which was associated with reduced oxidative stress and prevented doxorubicin-induced HF in mice [41]. Additionally, a combined theranostic nanoplatform combining Prussian blue and cerium oxide NPs demonstrated encouraging in vitro and in vivo results, including enhanced MRI, selective accumulation in inflamed cardiac tissue, and anti-inflammatory and anti-fibrotic effects that improve cardiac function [59].

#### 3.3.3. Sepsis-Induced Cardiomyopathy

Sepsis-induced cardiomyopathy (SICM) is a reduction in cardiac contractility resulting from increased pro-inflammatory cytokine release and endothelial and microvascular dysfunction due to a disseminated infectious cause [78]. MiR-133a is a microRNA molecule with recognized anti-apoptotic and anti-inflammatory properties, able to halt cardiac remodeling [79]. Hyaluronic acid–based NPs delivering miR-133a can effectively promote anti-inflammatory macrophage polarization and inhibit SOCS3/JAK/STAT3 signalling, reducing myocardial injury and inflammation in sepsis-induced cardiomyopathy animal models [42]. In addition, the NLRP3-inflammasome activation pathway in SICM can be targeted using macrophage membrane-coated NPs (MGP) to deliver recombinant human growth differentiation factor 15 (GDF15), a molecule with anti-inflammatory and cardioprotective effects, as suggested by Guo et al. in a murine model [43].

#### 3.3.4. Diabetic Cardiomyopathy

Diabetic cardiomyopathy is driven by metabolic abnormalities induced by hyperglycemia and insulin resistance, which promote mitochondrial dysfunction, oxidative stress, impaired calcium handling, and increased formation of advanced glycation end products. These processes trigger inflammatory signalling and cytokine release, leading to fibroblast activation, myocardial fibrosis, and progressive cardiac dysfunction that can ultimately result in heart failure [80,81]. Given the importance of early detection and targeted intervention, several nanotechnology-based strategies have been developed to identify and mitigate myocardial injury in diabetic cardiomyopathy. Liu et al. developed a dual-targeting nanoparticle probe based on SPIONs, containing a cardiac-homing peptide and an antibody against the fibrosis biomarker matrix metalloproteinase-2 (MMP2), enabling sensitive MRI detection of early myocardial fibrosis in diabetic mice [26]. Similarly, Du et al. designed a nanoplatform composed of extremely small iron oxide NPs functionalized with cardiac-homing and mitochondrial-targeting peptides to deliver Argonaute-2 to cardiac mitochondria, restoring mitochondrial function and reducing oxidative stress and inflammation in diabetic cardiomyopathy models [44].

### 3.4. Myocarditis and Pericarditis

Myocarditis is a disease involving inflammation of myocardial cells, most frequently due to viral infection, with non-infectious causes including autoimmune diseases, drugs, toxins, and genetic conditions [82]. Early detection can prevent complications, including arrhythmias, dilated cardiomyopathy and heart failure [83]. Current therapeutic approaches for acute myocarditis remain limited and include mostly supportive measures and guideline-directed HF therapies, highlighting the need for improved, etiology-specific treatment options that can efficiently suppress the immune response [84]. In this context, nanotechnology can facilitate targeted drug delivery and improve therapeutic bioavailability within inflamed cardiac tissue, with several researchers proposing different options based on experimental autoimmune myocarditis models. For example, a biomimetic hybrid membrane–coated NP loaded with the JAK1/2 inhibitor baricitinib enables targeted delivery to inflammatory sites, suppressing proinflammatory macrophage M1 polarization, pyroptosis, and oxidative stress [46]. In addition, a hybrid immune cell membrane–coated NP delivering siRNA against interferon regulatory factor-1 (IRF-1) has been shown to specifically target M1 macrophages and inhibit IRF1-mediated pyroptosis, thereby attenuating myocardial inflammation and disease progression in an experimental autoimmune myocarditis model [47]. Similarly, a protein G–conjugated nanomedicine (PSL-G) targets macrophages and promotes their shift from a pro-inflammatory to an anti-inflammatory phenotype, reducing cytokine production, macrophage infiltration, and myocardial fibrosis in another animal model, highlighting its potential as a therapeutic strategy for inflammatory heart diseases [48].

For pericarditis, the inflammation of the pericardial sac, nanotechnology-based approaches are very limited. Although infectious causes such as tuberculous pericarditis are still relevant in endemic regions, most cases in Western populations are idiopathic or recurrent and are increasingly recognized mechanistically as IL-1β-driven autoinflammatory conditions [82]. Current anti-inflammatory therapies, including colchicine and IL-1 inhibitors such as anakinra and rilonacept, have significantly improved the management of recurrent pericarditis, highlighting the importance of targeted immunomodulation in disease control [85]. In this context, nanotechnology-based drug delivery systems may represent a promising future strategy to enhance local anti-inflammatory efficacy while minimizing systemic adverse effects. Researchers from South Africa designed a platform based on macrophage-targeted mannan–chitosan NPs loaded with bedaquiline, a drug shown to shorten the duration of tuberculosis therapy, which has demonstrated efficient diffusion across human and porcine pericardia, representing a promising nanotechnology-based platform for localized drug delivery in tuberculous pericarditis [49]. Although nanomedicine applications in recurrent and non-infectious pericarditis remain largely unexplored, the successful use of NP-based anti-inflammatory delivery systems in other cardiovascular inflammatory diseases suggests potential future applications for targeted colchicine, corticosteroid, or IL-1 inhibitor delivery. However, these concepts remain speculative and require preclinical and clinical validation before application in clinical practice.

### 3.5. Atrial Fibrillation

Inflammation plays a central role in the initiation and progression of atrial fibrillation (AF). Several inflammatory mediators, including IL-6, TNF-α, and NF-κB, have been associated with the induction of electrical remodeling and increased fibroblast activation, ultimately leading to fibrosis and structural remodeling of atrial tissue [86]. Moreover, activation of the NLRP3 inflammasome in atrial cardiomyocytes, driven by chronic inflammatory states such as diabetes, obesity and hypertension, may further promote adverse atrial structural remodeling and contribute to increased AF susceptibility [87,88].

Despite the recognized contribution of inflammation to AF pathogenesis, anti-inflammatory therapeutic strategies specifically targeting AF remain insufficiently explored, and nanotechnology-based approaches are still limited. However, lipid NPs, polymeric systems (such as PLGA/PEG), and metal-based NPs have been investigated as carriers for targeted delivery of antifibrotic drugs in other cardiac and non-cardiac conditions. Therefore, it may be hypothesized that similar approaches could be applied in AF therapy, although further validation is required [89]. Similarly, nanoplatforms capable of modulating NLRP3 inflammasome activation have shown promising anti-inflammatory effects in preclinical cardiovascular studies and may represent a future therapeutic direction in AF [90]. However, direct evidence in AF-specific experimental or clinical settings remains scarce, and further validation is required before clinical translation.

NPs may also enhance the efficacy of catheter ablation by reducing local inflammation and potentially preventing early AF recurrence. In a porcine model, polylactic-co-glycolic acid NPs loaded with budesonide were delivered locally during radiofrequency ablation procedures and demonstrated immediate heat-triggered and sustained myocardial anti-inflammatory effects. This approach was associated with reduced local hematoma formation, inflammatory cell infiltration, and IL-6 levels in this animal model [50].

Beyond synthetic NPs, naturally derived nanosized extracellular vesicles, such as exosomes, have also emerged as important mediators in AF pathophysiology. Exosomal microRNAs can regulate key processes involved in atrial remodeling, including fibrosis, inflammation and cardiomyocyte apoptosis. For example, atrial myocyte-derived exosomes deficient in miR-23a through lncRNA NRON suppression were shown to promote anti-inflammatory M2 macrophage polarization and decrease fibrosis markers in atrial fibroblasts, suggesting a potential mechanism for targeting inflammatory remodeling in AF [91,92].

Together, these findings suggest that nanomedicine-based anti-inflammatory approaches may emerge as adjunctive strategies for AF management, particularly in combination with interventional therapies such as catheter ablation. Nevertheless, the current evidence base remains predominantly preclinical and further AF-specific translational studies are required.

### 3.6. Vascular Diseases and Venous Thromboembolism

Vascular inflammation through endothelial cell activation and macrophage polarization towards the pro-inflammatory type is a key contributing factor to the structural changes in the arterial wall that eventually lead to abdominal aortic aneurysm (AAA) formation. Various nanosystems have been implemented in the imaging and treatment of AAA [93]. USPIO (Ultrasmall Superparamagnetic Iron Oxide) and SPIONs targeting macrophages and neutrophils in the arterial wall have shown promising results when used as imaging probes in MRI, while gold NPs have been used in molecular imaging to directly visualize extracellular matrix components, such as MMP activation and fibrin [27,28,29,30]. Delivery of anti-inflammatory agents in the aortic wall through nanocarriers has also been explored. Examples include NPs conjugated with pitavastatin and miR-223, and results have demonstrated a reduction in macrophage concentration and a shift in their polarization towards the M2 (anti-inflammatory) phenotype, thus halting disease progression and potentially preventing rupture of AAA [51,52].

Nanomedicine-based approaches are also emerging to suppress inflammation associated with venous thromboembolism by enabling targeted intervention within the inflammatory microenvironment. Nanoplatforms based on liposomes, polymeric and inorganic NPs have demonstrated promising results in targeting inflammatory molecules, activated platelets, endothelial and immune cells, all of which play a crucial role in endothelial dysfunction, thrombus formation and de-stabilization [94]. Beyond traditional thrombolytic therapies for the treatment of deep vein thrombosis (DVT), researchers have developed DNA nanostructures that reduce programmed endothelial cell death, oxidative stress, and inflammation by modulating immune-inflammatory pathways associated with vascular wall dysfunction and increased thrombosis risk [53].

## 4. Emerging Nanoplatforms

Recent advances in nanotechnology-based approaches to cardiovascular inflammation include a shift towards more biocompatible, multifunctional, and disease-specific nanosystems, particularly in treatment options. Among these, biomimetic NPs represent a breakthrough, offering tissue-specific delivery and enhanced therapeutic efficacy. These NPs, often derived from cell membranes or native tissues, exhibit improved biocompatibility, immune evasion, and intrinsic bioactivity, enabling more effective targeting of pathological processes such as inflammation, oxidative stress, and apoptosis following myocardial injury [95]. In this context, tissue-derived nanoplatforms are being integrated with advanced biomaterials to create multifunctional systems that closely resemble the native cardiac environment. For example, adipose-derived active NPs (ADANs), which are nanospheres enriched in adipokines, have been incorporated into a conductive, hydrogel-based 3D-printed cardiac patch (Bio-GPP@A) with properties similar to those of cardiac tissue. This patch has demonstrated a significant reduction in inflammation, oxidative stress, and myocardial cell apoptosis post-MI in an animal model, highlighting the therapeutic potential of biomimetic NPs in improving cardiac function and reversing LV remodeling [38]. In addition, HDL biomimetic NPs that mimic the structure and function of native HDL are increasingly being explored as therapeutic options for atherosclerosis. Evidence has shown that these NPs may enhance cholesterol efflux, reduce vascular inflammation and promote atheroprotective effects by leading to plaque regression [96].

Complementing these biomimetic strategies, catalytic nanoplatforms such as nanozymes have emerged as another promising therapeutic approach. These engineered NPs exhibit intrinsic enzyme-like activities and exert anti-inflammatory effects by reducing ROS-driven oxidative stress and modulating immune responses [97]. In ASCVD, nanozymes can mimic natural antioxidant enzymes such as catalase to regulate oxidative stress and inflammation, while also enabling targeted and controlled delivery of therapeutic agents. Preclinical studies show that nanozyme-based systems can reduce ROS-driven endothelial dysfunction, limit lipid peroxidation and ultimately improve plaque stability [98]. Additionally, since mitochondrial dysfunction remains the central part of I/R injury in MI, a mitochondria-targeting engineered ferritin nanocage nanozyme (imFTn-Ru) has been recently developed to catalyze NO production, thereby reducing mitochondrial ROS, inhibiting permeability transition pore opening, and protecting cardiomyocytes against I/R injury through organelle-specific delivery and activity [39].

Beyond synthetic and inorganic nanozyme platforms, biologically derived nanovesicles such as exosomes have recently gained increasing attention in cardiovascular nanomedicine. These endogenous vesicles provide cell-free alternatives with superior biocompatibility and reduced immunogenicity. Examples of their anti-inflammatory potential include microRNA delivery that promotes M2 macrophage polarization in ASCVD, dilated cardiomyopathy and myocardial I/R injury [31,45,99].

## 5. Challenges and Future Perspectives

### 5.1. Clinical Translation, Biological Barriers, Manufacturing and Regulatory Issues

Even though nanomedicine has shown considerable promise for diagnosing and treating cardiovascular inflammation, several translational issues must be addressed before it can be integrated into clinical practice. Safety concerns, including potential immunogenicity, cytotoxicity, and long-term biodistribution and clearance profiles, remain incompletely characterized, particularly in repeated, long-term use [100]. In addition, physiological and pathological biological processes significantly constrain translational efficiency, including rapid reticuloendothelial system clearance, insufficient myocardial accumulation, endothelial dysfunction, and highly dynamic inflammatory microenvironments that may interfere with targeted delivery [101]. To overcome these challenges and enhance the EPR effect in cardiac tissues, several approaches have been explored, including optimizing the size and shape of NPs to better match the biological characteristics of cells, and active targeting, where NPs are directed towards specific receptors [101].

Nanomedicine applications for cardiovascular inflammation face significant manufacturing and regulatory hurdles. Manufacturing is complicated by the difficulty of producing NPs with consistent size, surface charge, and drug-loading capacity at scale while maintaining batch-to-batch reproducibility, which is critical since these physicochemical properties are directly linked to biodistribution and therapeutic efficacy. Lipid NPs, polymeric carriers, biomimetic platforms, and stem cell-based and exosome-based therapies each present distinct manufacturing challenges; for example, membrane-coating procedures used in biomimetic NPs are difficult to standardize and scale without compromising the biological activity of the surface coating [102,103]. On the other hand, regulatory challenges derive from the heterogeneous nature of nanomedicines and the lack of standardized protocols to assess their quality, creating uncertainty for FDA and EMA approvals. The absence of globally acceptable regulatory frameworks may further impact innovation, research and clinical translation of nanomedicine [104].

However, perhaps one of the main limitations of nanomedicine-based applications in CVD is that most data derive from in vitro or in vivo animal studies. In the diagnosis field, early clinical evidence has been less encouraging than preclinical studies, with one trial reporting no significant difference in USPIOΝ uptake between patients with myocarditis and healthy controls, underscoring the limitations of current targeting strategies and the gap between animal models and human disease [105]. In addition, as discussed previously, most therapeutic applications, including biomimetic NPs, drug-loaded nanocarriers, and targeted mRNA NPs, remain in preclinical development despite promising results in animal models.

### 5.2. Future Research, Artificial Intelligence and Precision Nanomedicine

Future research should focus on shifting from experimental nanoplatforms to clinically applicable, disease-specific systems. Large-scale, interdisciplinary clinical trials should be conducted to evaluate the efficacy and safety of these platforms, in addition to their diagnostic and therapeutic potential.

The role of artificial intelligence (AI) in expanding the potential of nanomedicine in CVD is a rapidly evolving area. Several applications have been described, including AI-driven selection of optimal materials, NP size and formulation, accurate prediction of NP pharmacokinetics, biodistribution, and interactions with human tissues, as well as potential toxicity [106,107]. Through computational pharmaceutics, which includes the integration of AI in pharmacotherapy, researchers are able to predict drug activity, in vitro release, stability, distribution and in vivo pharmacokinetic parameters, therefore optimizing drug delivery [108]. In the diagnostic domain, AI algorithms have been explored to enhance the sensitivity and specificity of NP-based imaging by improving signal-to-noise discrimination and enabling faster, lower-cost interpretation of results [109]. In addition, machine learning approaches, including supervised, unsupervised, and deep learning models, have been increasingly applied in nanomedicine to optimize NP design and synthesis, enhance molecular imaging, and improve drug delivery accuracy. However, the application of AI in nanomedicine is limited by several issues, including data interpretability and “black-box” challenges, lack of standardized datasets, algorithm selection and validation issues, as well as concerns regarding model transparency and regulatory oversight [109].

Collectively, these advances point toward a transition from experimental nanoplatforms to precision nanomedicine, where the integration of biomimetic design, stimuli-responsive behaviour, and AI-guided optimization may enable the development of clinically relevant, patient-specific therapies for cardiovascular inflammation.

## 6. Conclusions

Inflammation is a widely recognized mechanism for the initiation and progression of CVD, highlighting the need for more precise diagnostic and therapeutic strategies. In this context, nanomedicine may offer several advantages over conventional approaches by enabling targeted drug delivery, enhanced molecular imaging, improved biodistribution, and the development of theranostic platforms capable of integrating diagnostic and therapeutic functions.

Research has focused primarily in the fields of atherosclerosis and myocardial infarction, where nanotechnology-based imaging probes and targeted anti-inflammatory delivery systems have shown promising preclinical and early translational results. In contrast, applications in heart failure, cardiac arrhythmias and myopericardial diseases remain highly exploratory. Although several nanoplatforms have shown encouraging anti-inflammatory and cardioprotective effects in experimental models, the majority of available data remain preclinical.

Despite these encouraging results, important limitations remain, particularly regarding safety, toxicity, biodistribution, and clinical application of nanomedicine in cardiovascular inflammation. With recent advances, including biomimetic NPs and stem cell–derived or exosome-based systems, the potential of nanotechnology has been expanded with improved efficiency and biocompatibility. In addition, the role of AI-based solutions is still investigational, but may further improve precision medicine and facilitate individualized therapeutic strategies. However, larger translational and clinical studies are required to establish the safety, efficacy, and clinical applicability of these emerging technologies before implementation in routine cardiovascular practice.

## Figures and Tables

**Table 1:** Nanotechnology-based diagnostic platforms in cardiovascular inflammation.

Disease	Nanoplatform	Imaging Modality	Target	Model	Main Finding	Translation Stage	Reference
Atherosclerosis	USPIO/SPIONs	MRI	Macrophages	Animals/Human	Detection of vascular inflammation and vulnerable plaques	Preclinical and early clinical	[8,9]
Atherosclerosis	Gd-DTPA encapsulating hydrogen HPs (Ab36-cHANPs)	MRI	CD36-positive plaque-associated macrophages	Ex vivo human plaque model	High risk plaque	Preclinical	[10]
Atherosclerosis	Gd(III)-doped amorphous calcium carbonate NPs	MRI	Microcalcifications (alendronate)/inflammation (trimannose)	Murine	Inflammatory plaque burden	Preclinical	[11]
Atherosclerosis	NPs with iodixanol and hydrophobic moieties	CT	Macrophages (CD68+)	Rabbit	Macrophage-rich plaque detection	Preclinical	[12]
Atherosclerosis	99mTc-Gold NPs-Annexin V	SPECT/CT	Apoptotic cells	ApoE−/− murine	Vulnerable plaque detection	Preclinical	[13]
Atherosclerosis	64Cu-vMIP-II-comb NPs	PET/CT	Macrophage chemokine receptors	ApoE−/− murine	Plaque inflammation and progression	Preclinical	[14]
Atherosclerosis	64Cu-DAPTA-Comb NPs	PET/CT	CCR5 chemokine receptor	ApoE−/− murine	Plaque inflammation and progression	Preclinical	[15]
Atherosclerosis	ICAM-1-targeted microbubbles	IVUS	ICAM-1	Rabbit	Early atherosclerosis imaging	Preclinical	[16]
Atherosclerosis	Porphyrin lipid NPs	OCT-fluorescence imaging	CD68+ macrophages	ApoE−/− murine	Early atherosclerosis imaging	Preclinical	[17]
Atherosclerosis	Infrared-emitting semiconductor quantum dots (QDs)	OCT-fluorescence imaging	Structural plaque characterization	Ex vivo rabbit arterial model	Plaque characterization	Preclinical	[18]
Myocardial infarction	USPIONs	MRI	Myocardial inflammatory cells/macrophages	Human	Inflammatory activity	Early clinical	[19,20]
Myocardial infarction	Modified polyglucose NPs (^18^F-Macroflor)	PET	Macrophages	Murine and rabbit	Inflammatory burden in infarct and plaque	Preclinical	[21]
Myocardial infarction	CLIO-AF647 NPs	MRI/optical	CCR2+ inflammatory myeloid cells	Murine	Post-MI inflammation and prediction of adverse remodeling	Preclinical	[22]
Myocardial infarction	Angiotensin II-functionalized Ag_2_S nanodots	NIR-II	AT1 receptor	Murine	Ischemic area visualization	Preclinical	[23]
Myocardial infarction	^19^F-HDL nanotracer	^19^F-MRI/PET/optical imaging	Myeloid cell activity	Murine	Systemic inflammation responses	Preclinical	[24]
Myocardial infarction	Functionalized MnO-PEG-Cy5.5 NPs	MRI/NIR	Infarcted myocardium	Rodent	Infarct area visualization	Preclinical	[25]
Diabetic cardiomyopathy	MMP2-targeted SPIONs	MRI	MMP2	Murine	Early detection of myocardial fibrosis	Preclinical	[26]
Abdominal aortic aneurysm	Elastin antibody-conjugated gold NPs (EL-GNPs)	CT	Degraded elastin/ECM	Murine	CT signal correlated with elastin degradation and rupture risk	Preclinical	[27]
Abdominal aortic aneurysm	SPIONs	MRI/FLI/CT	Ly6G-positive neutrophils, CXCR4	Murine	Multimodal imaging of neutrophil infiltration and AAA severity	Preclinical	[28,29,30]

**Table 2:** Therapeutic nanoplatforms targeting cardiovascular inflammation.

Disease	Nanoplatform	Therapeutic Agent	Target	Model	Therapeutic Effect	Translation Stage	Reference
Atherosclerosis	Stem-cell derived exosomes	Exosome-mediated immunomodulation	Macrophage polarization and plaque inflammation	Murine	Reduced plaque burden and macrophage infiltration, promoted M2 polarization	Preclinical	[31]
Myocardial infarction	NM@PDA@PU biomimetic NPs	Puerarin	Pyroptotic cardiomyocytes, inflammatory macrophages	Murine	Reduced pyroptosis, improved cardiac remodeling and function	Preclinical	[32]
Myocardial infarction	ColCaNPs (calcium carbonate NPs)	Colchicine	Inflammatory macrophages/TLR4-NFκB-NLRP3 pathway	Rodent	Reduced infarct size, fibrosis, pyroptosis, and inflammatory cytokine expression	Preclinical	[33]
Myocardial infarction	PTPN biomimetic NPs	tPA + protocatechualdehyde (PC)	Coronary thrombus and ROS-mediated reperfusion injury	Murine	Targeted thrombolysis, reperfusion injury reduction, and mitochondrial protection	Preclinical	[34]
Myocardial infarction	VB@MOF/TA mitochondria-targeted NPs	Verbascoside (VB)	Mitochondrial oxidative stress, inflammatory macrophages	Rodent	Reduced cardiomyocyte apoptosis, modulation of macrophage polarization, improved cardiac remodeling and function	Preclinical	[35]
Myocardial infarction	PUE@TK/CHP-L dual-modified liposomes	Puerarin	Ischemic cardiomyocytes and ROS-rich microenvironment	Murine	Reduced apoptosis and ferroptosis, enhanced targeting of ischemic myocardium, attenuation of MI/RI	Preclinical	[36]
Myocardial infarction	Transferrin-conjugated pH-responsive nanomicelles	MCC950	NLRP3 inflammasome/TFR1	Rodent	Reduced inflammasome activation and improved survival after I/R injury	Preclinical	[37]
Myocardial infarction	conductive, hydrogel-based 3D-printed cardiac patch (Bio-GPP@A)	ADAN-mediated anti-inflammatory and regenerative therapy	Oxidative stress, inflammation, and post-MI remodeling	Murine	Reduced ROS production and apoptosis, promoted angiogenesis, attenuated ventricular remodeling, improved cardiac function	Preclinical	[38]
Myocardial infarction	imFTn-Ru nanozyme (engineered ferritin nanocage)	Mitochondria-targeted NO-generating nanozyme therapy	Mitochondrial oxidative stress and dysfunction	Murine/in vitro	Attenuated I/R injury	Preclinical	[39]
HF	SPION-loaded NO-releasing hydrogel NPs (NO-RPs)	Nitric oxide (NO)	Endothelial dysfunction and inflammatory pathways	Zebrafish HF model	Improved cardiac function and blood flow, reduced IL-6 and COX-2 expression	Preclinical	[40]
Doxorubicin cardiomyopathy	TPCD-based NPs	ROS-scavenging TPCD material ± Ac2-26 peptide	ROS pathways	Murine	Reduced oxidative stress and inflammation, improved cardiac function	Preclinical	[41]
Septic cardiomyopathy	HA-based nanogel	miR-133a	SOCS3/JAK/STAT3 signaling and macrophage polarization	Rodent	Reduced inflammation and cardiomyocyte apoptosis, promoted M2 macrophage polarization	Preclinical	[42]
Septic cardiomyopathy	Macrophage membrane-coated PLGA NPs (MGP)	Recombinant human GDF15 (rhGDF15)	NLRP3 inflammasome/GDF15-MYPT1-YBX-1 pathway	Murine	Reduced inflammation and oxidative stress, improved left ventricular function and contractility	Preclinical	[43]
Diabetic cardiomyopathy	ESC-AGO-2 NPs(ESIO-SS-31-CHP)	Argonaute-2 (AGO-2)	Mitochondrial function	Murine	Restored mitochondrial homeostasis, reduced oxidative stress and inflammation, improved mitochondrial function	Preclinical	[44]
Dilated cardiomyopathy	Mesenchymal stem cell-derived exosomes (MSC-Exos)	Exosome-mediated immunomodulation	Macrophage polarization (JAK2/STAT6 pathway)	Murine	Improved cardiac function, reduced inflammation, cardiomyocyte apoptosis, and adverse remodeling	Preclinical	[45]
Autoimmune Myocarditis	BM@[RAW-EL4] biomimetic NPs	Baricitinib	Macrophages, JAK2/STAT1 signaling, pyroptosis	Murine	Reduced inflammatory infiltration, macrophage polarization, pyroptosis, and myocardial injury	Preclinical	[46]
Autoimmune Myocarditis	siIRF1@ZIF@HM NPs (T lymphocyte–macrophage hybrid membrane-coated ZIF-8)	siIRF1 delivery	IRF1-mediated macrophage pyroptosis	Murine	Reduced myocardial inflammation, inhibited macrophage pyroptosis, attenuated myocarditis progression	Preclinical	[47]
Autoimmune Myocarditis	Protein G-conjugated bioinspired NPs (PSL-G)	PSL-G anti-inflammatory nanomedicine	Macrophages (M1 → M2 polarization)	Murine	Reduced myocardial inflammation, macrophage infiltration, and fibrosis	Preclinical	[48]
Tuberculous pericarditis	Mannan-chitosan NPs	Bedaquiline	Pericardial macrophages	Human/Porcine	Localized anti-inflammatory therapy	Early clinical/Preclinical	[49]
Atrial fibrillation	PLGA NPs	Budesonide	NLRP3/fibrosis pathways	Porcine	Reduced hematoma, inflammation and IL-6 levels	Preclinical	[50]
Abdominal aortic aneurysm	Pitava-NPs (PLGANPs)	Pitavastatin	Monocytes/macrophages and MCP-1-mediated inflammation	Murine	Reduced macrophage accumulation, MMP activity, elastin degradation, and AAA progression	Preclinical	[51]
Abdominal aortic aneurysm	Galactose-modified miR-223-loaded NPs (MirNPs)	miR-223	Macrophage polarization and NLRP3 inflammasome	Murine	Promoted M2 macrophage polarization, reduced inflammation and AAA progression	Preclinical	[52]
Deep vein thrombosis	Tetrahedral DNA nanostructures (TDNs)	TDNs	Endothelial necroptosis (RIP3/MLKL pathway), oxidative stress, and inflammation	Murine	Reduced thrombus formation, endothelial injury, oxidative stress, and inflammatory signaling	Preclinical	[53]

**Table 3:** Nanotechnology-based theranostic platforms in cardiovascular inflammation.

Disease	Nanoplatform	Diagnostic Modality	Therapeutic Component	Model	Main Finding	Translation Stage	Reference
Atherosclerosis	Gd/arginine-polydopamine NPs (AGPDAR-146a)	MRI	miR-146a	Murine	Plaque imaging and anti-inflammatory effect for stabilization	Preclinical	[54]
Atherosclerosis	ROS/MMP-responsive π-conjugated polymer NPs (PLCDP@PMH)	PAI	LCDP complex (liver X receptor ligand T0901317, prednisolone, β-cyclodextrin)	Murine	Targeted therapy with real-time plaque visualization	Preclinical	[55]
Atherosclerosis	Cyanine probe (MMR-Lobe-Cy)	OCT-NIRF	PPARγ agonist (lobeglitazone)	Rabbit	Plaque stabilization and molecular imaging	Preclinical	[56]
Myocardial infarction	Liposomes, metal-polyphenol network (MPN) film, hybrid immune-cell membrane (HM4oRL)	MRI/fluorescence	4-Octyl itaconate (4-OI)	Murine	Anti-pyroptotic effects	Preclinical	[57]
Myocardial infarction	Perfluorocarbon NPs	Fluorescent microscopy/19F MRI	PPACK (thrombin inhibitor)	Rodent	Local thrombin suppression, reduction in inflammation, microvascular injury and I/R injury	Preclinical	[58]
Anthracycline-induced cardiomyopathy	Prussian blue NPs (PB@CeO_2_ NPs)	MRI	cerium oxide (CeO_2_) NPs	Murine	Antioxidative and anti-inflammatory effects	Preclinical	[59]

## Data Availability

All data generated in this research are included in the article.
